# Reply to Itagaki et al. Comment on “Dorobisz et al. Assessment of Prognostic Factors, Clinical Features Including the Microbiome, and Treatment Outcomes in Patients with Cancer of Unknown Primary Site. *Cancers* 2024, *16*, 3416”

**DOI:** 10.3390/cancers16233952

**Published:** 2024-11-26

**Authors:** Karolina Dorobisz, Tadeusz Dorobisz, Katarzyna Pazdro-Zastawny

**Affiliations:** 1 Department of Otolaryngology, Head and Neck Surgery, Wrocław Medical University, Borowska 213, 50-556 Wroclaw, Poland; 2 Department of Vascular, General and Transplantation Surgery, Wroclaw Medical University, Borowska 213, 50-556 Wroclaw, Poland

In his commentary [1] to our article “Assessment of Prognostic Factors, Clinical Features Including the Microbiome, and Treatment Outcomes in Patients with Cancer of Unknown Primary Site Cancers” published in the journal *Cancers* [2], Tatsuki et al. states that when analyzing the percentage of the microbiome, when one component increases, another decreases. Of course, this is true; the reduction in diversity and dominance of certain groups of bacteria or their genes causes dysbiosis, while reducing the number of other types of bacteria. The paper also presents the results of microbiological cultures, showing how significantly the results differ between them. The paper in the discussion presents many publications where the view on the microbiome is similar, and the analyses are consistent with the one used in our work. Comparing the dominance of a bacterial group and its percentage share provides a basis for drawing conclusions. Of course, we agree with the authors that a different analysis can be performed. However, we chose to present dysbiosis as a whole flora composition, which in our opinion is best for showing pathology and differences between groups.

## References

[1] Itagaki T., Kasai M., Sakata K.-i., Hasebe A. (2024). Comment on Dorobisz et al. Assessment of Prognostic Factors, Clinical Features Including the Microbiome, and Treatment Outcomes in Patients with Cancer of Unknown Primary Site. *Cancers* 2024, *16*, 3416. Cancers.

[2] Dorobisz K., Dorobisz T., Pazdro-Zastawny K. (2024). Assessment of Prognostic Factors, Clinical Features Including the Microbiome, and Treatment Outcomes in Patients with Cancer of Unknown Primary Site. Cancers.

